# Dustless and Washable Hospitals

**Published:** 1923-11

**Authors:** Ralph B. Cannings

**Affiliations:** Secretary. City of London Maternity Hospital, City Road, E.C. 1


### "Dustless and Washable Hospitals."

SIR,—Referring to the article in your October issue on the "Dustless and Washable Hospitals," it may interest you to know that for many years past the City of London Maternity Hospital has been decorated throughout with Robbialac Enamel Paint, with very satisfactory results.

RALPH B. CANNINGS,

Secretary.

City of London Maternity Hospital,

City Road, E.C. 1.

### In Defence of Vaccination.

The Research Defence Society (11, Chandos Street, Cavendish Square) have published, at the price of sixpence, the address on "Smallpox and Vaccination," delivered to members of the House of Commons by Mr. John McVail last July. The address makes out a very strong case for vaccination, and should be valuable for distribution among those who are opposed or indifferent to its practice. The author suggests that the Government should draw "some practical distinction between a parent no doubt wrong-headed yet sincere in his conviction that vaccination is evil, and another parent who is merely indifferent or neglectful.

